# Lifetime ovulatory years and ovarian cancer gene expression profiles

**DOI:** 10.1186/s13048-022-00995-1

**Published:** 2022-05-13

**Authors:** Naoko Sasamoto, Paul A. Stewart, Tianyi Wang, Sean J. Yoder, Srikumar Chellappan, Jonathan L. Hecht, Brooke L. Fridley, Kathryn L. Terry, Shelley S. Tworoger

**Affiliations:** 1 Department of Obstetrics and Gynecology, Brigham and Women’s Hospital and Harvard Medical School, 221 Longwood Avenue, Boston, MA 02115 USA; 2 Department of Biostatistics and Bioinformatics, H. Lee Moffitt Cancer Center and Research Institute, Tampa, FL USA; 3 Department of Cancer Epidemiology, H. Lee Moffitt Cancer Center and Research Institute, Tampa, FL USA; 4 Molecular Genomics Core, H. Lee Moffitt Cancer Center and Research Institute, Tampa, FL USA; 5 Department of Tumor Biology, H. Lee Moffitt Cancer Center and Research Institute, Tampa, FL USA; 6 Department of Pathology, Beth Israel Deaconess Medical Center and Harvard Medical School, Boston, MA USA; 7 Department of Epidemiology, Harvard T.H. Chan School of Public Health, Boston, MA USA

**Keywords:** Ovulatory years, Gene expression, Ovarian cancer tumor, RNA sequencing, Carcinogenesis

## Abstract

**Background:**

Greater ovulatory years is associated with increased ovarian cancer risk. Although ovulation leads to an acute pro-inflammatory local environment, how long-term exposure to ovulation impacts ovarian carcinogenesis is not fully understood. Thus, we examined the association between gene expression profiles of ovarian tumors and lifetime ovulatory years to enhance understanding of associated biological pathways.

**Methods:**

RNA sequencing data was generated on 234 invasive ovarian cancer tumors that were high-grade serous, poorly differentiated, or high-grade endometrioid from the Nurses’ Health Study (NHS), NHSII, and the New England Case Control Study. We used linear regression to identify differentially expressed genes by estimated ovulatory years, adjusted for birth decade and cohort, overall and stratified by menopausal status at diagnosis. We used false discovery rates (FDR) to account for multiple testing. Gene set enrichment analysis (GSEA) with Cancer Hallmarks, KEGG, and Reactome databases was used to identify biological pathways associated with ovulatory years.

**Results:**

No individual genes were significantly differentially expressed by ovulatory years (FDR > 0.19). However, GSEA identified several pathways that were significantly associated with ovulatory years, including downregulation of pathways related to inflammation and proliferation (FDR < 1.0 × 10^–5^). Greater ovulatory years were more strongly associated with downregulation of genes related to proliferation (e.g., E2F targets, FDR = 1.53 × 10^–24^; G2M checkpoints, FDR = 3.50 × 10^–22^) among premenopausal versus postmenopausal women at diagnosis. The association of greater ovulatory years with downregulation of genes involved in inflammatory response such as interferon gamma response pathways (FDR = 7.81 × 10^–17^) was stronger in postmenopausal women.

**Conclusions:**

Our results provide novel insight into the biological pathways that link ovulatory years to ovarian carcinogenesis, which may lead to development of targeted prevention strategies for ovarian cancer.

**Supplementary Information:**

The online version contains supplementary material available at 10.1186/s13048-022-00995-1.

## Introduction

Greater lifetime ovulatory years has been consistently associated with increased ovarian cancer risk in both premenopausal and postmenopausal women [1–8]. A large international consortium reported that ovarian cancer risk increased by 14% per 5-year increase in lifetime ovulatory years, with median lifetime ovulatory years in this study being 36.3 [5]. This association was strongest for serous, endometrioid, and clear cell tumors, but not other histologies [5]. These observations together with the abundant evidence on reproductive factors that lead to reduced number of ovulatory years, such as parity and oral contraceptive use, are consistently associated with decreased ovarian cancer risk [9] support that incessant ovulation may be a causal mechanism underlying ovarian carcinogenesis [10, 11].

Ovulation involves follicular rupture and injury to the ovarian epithelium, resulting in production of inflammatory mediators and reactive oxidants that cause DNA damage [12–14]. Accumulating exposure to the pro-inflammatory local environment and wound repair that follows ovulation at the ovarian surface and fallopian tube are also thought to contribute to ovarian carcinogenesis. However, the biological mechanisms of the association between long term exposure to ovulation and ovarian cancer risk are not fully understood. Thus, we examined the association between gene expression profiles (transcriptome) of ovarian cancer tumors and lifetime ovulatory years, overall and by menopausal status at diagnosis, to enhance understanding of the underlying carcinogenic pathways, focusing on high-grade serous, poorly-differentiated, and high-grade endometrioid tumors, which together are classified as type II ovarian tumors [15].

## Materials and methods

### Study population and assessment of ovulatory years

#### Nurses’ Health Studies (NHS/NHSII)

The Nurses’ Health Study (NHS) is a U.S.-based prospective cohort study established in 1976 enrolling 121,700 female nurses aged 30–55 years and the Nurses’ Health Study II (NHSII) was established in 1989, enrolling 116,429 female nurses aged 25–42 [16, 17]. All participants were followed biennially via questionnaires that assessed updated information on lifestyle and reproductive exposures as well as medical history. Once a participant reported having ovarian cancer or was found to have died of ovarian cancer via the National Death Index, diagnosis was confirmed in 94% of the cases by pathology reports. Other cases were confirmed by linkage to tumor registries. A pathologist reviewed the associated records to confirm the diagnosis and abstract clinical information on stage. Ovarian tumor formalin fixed paraffin embedded (FFPE) tissue blocks were collected from confirmed ovarian cancer cases, which has been previously described, and centrally reviewed by single gynecologic pathologist (JH), who assessed histology and grade [18, 19]. After including cases determined to be high-grade serous, poorly differentiated, or high-grade endometrioid, there were blocks available from 209 cases (157 from NHS and 52 from NHSII) diagnosed from 1995 to 2013; cases diagnosed before 1995 were excluded due to poor RNA quality/quantity upon extraction. Lifetime ovulatory years were calculated as the difference of age at menopause for postmenopausal women or age at blood collection for premenopausal women minus age at menarche, subtracting duration of OC use and one year per pregnancy based on covariate data collected up to 2 years prior to diagnosis.

#### New England Case Control study (NEC)

The New England Case Control Study (NEC) is a population-based case control study of ovarian cancer enrolling participants aged 18–80 years from New Hampshire and Eastern Massachusetts over three phases (1992–1997, 1998–2002, 2003–2008) [20, 21]. Briefly, 2,203 (71%) of eligible cases participated in the study that were identified using registries of area hospitals. In-person interviews collected detailed information on lifestyle and reproductive exposures as well as medical history that occurred up to one year before diagnosis. Confirmation of ovarian cancer diagnosis along with abstraction of stage was done through surgical and pathological report review. FFPE ovarian tumor tissue blocks were collected from confirmed ovarian cancer cases, as described previously; cases were reviewed centrally by one pathologist (JH) to assess histology and grade [18, 22]. After including cases determined to be high-grade serous, poorly differentiated, or high-grade endometrioid, blocks were available from 109 cases diagnosed from 1995 to 2008. Lifetime ovulatory years was calculated as in NHS/NHSII based on data assessed up to one year prior to ovarian cancer diagnosis.

### RNA extraction and sequencing

We extracted RNA and DNA simultaneously from 1.5 mm diameter tumor tissue cores taken from FFPE tissue blocks from areas of tumor (circled by the pathologist) of treatment naïve primary ovarian tumors using the Qiagen All-Prep RNA isolation kit. The Illumina TruSeq™ RNA Exome Library Preparation Kit (Illumina Inc., San Diego, CA) was used to prepare the RNA-sequencing libraries, following the manufacturer’s protocol. In brief, 100 ng of RNA with DV200 > 15% was used as input into the RNASeq library preparation. Then, 200 ng of each sample cDNA library were normalized, pooled in four sample groups, and enriched twice with the Illumina probes. The libraries were sequenced on multiple NextSeq 500 High-Output 150 cycle sequencing runs to target an average of 25 million pairs of 75-base reads per sample. Following initial quality assessment and adaptor trimming, sequencing reads were mapped against human reference genome hs37d5 using STAR-2.5.3a [23]. Quantification of read counts aligned to the region associated with each gene was performed using HTSeq 0.6.1 [24] based on Gencode29 gene model. Read counts of all samples were normalized using the median-of-ratios method implemented in R/Bioconductor package DESeq2 v1.6.3 [25]. Two biological replicates were processed in technical triplicate to measure the reproducibility of the RNA-seq results. Supplementary Figure S1 shows extremely high correlation (Spearman’s R = 0.98–0.99) for both sets of technical replicates.

After RNA extraction, the samples were reviewed for quality on the Agilent TapeStation 4200 RNA ScreenTape following the recommendations described in the Illumina Technical Note [26]. Of the 323 tumor cores that had RNA and DNA simultaneously extracted, 35 (11%) samples were removed due to poor RNA quality defined as having DV200 values ≤ 15%. We attempted to conduct RNA sequencing on 288 extracted RNA samples (including 4 replicate samples) and successfully generated data on 253 (122 from NHS, 45 from NHSII, 86 from NEC) samples for bioinformatics analysis. Among these, we used 234 type II ovarian tumor samples (108 from NHS, 43 from NHSII, 83 from NEC) that had data on lifetime ovulatory years.

### RNA-seq data analysis

The *haven* package, part of the R Tidyverse [27], was used to read in clinical data from*.sas7bdat* format. The function *filterByExpr()* from the R package *edgeR* was used to filter genes with low expression [28], resulting in 14,483 genes for further analysis. We applied Trimmed Mean of M values (TMM) normalization using *calcNormFactors()*, also from *edgeR*, to account for differences in library size between samples. We next transformed the data using *voom* [29, 30]. We determined that no batch or study effects were present using principal component analyses, so we pooled the data from NHS, NHSII, and NEC (Supplementary Figure S2). We fit linear models of gene expression and lifetime ovulatory years (continuous) using *limma*, adjusting for decade of birth and study site [31]. We used the Benjamini–Hochberg false discovery rate (FDR) to account for multiple testing. We performed gene set enrichment analysis with *fgsea* using the log fold changes from *limma* as the ranks with Cancer Hallmarks, KEGG, and Reactome databases to identify biological pathways associated with ovulatory years [32, 33]*.* Enrichment Scores (ES) represent the degree of overrepresentation of genes in a gene set at the top or bottom of a ranked list of genes. ES are linked to the gene set sizes and correlations between gene sets and the gene expression data. To allow for the direct comparison of results across gene sets, Normalized Enrichment Scores (NES) were calculated [33]. FDR *p*-values were calculated with the Benjamini–Hochberg procedure. Using the R library *ComplexHeatmap* [34], we created heatmaps including individual genes within the significant inflammation/immune pathways and proliferation-related pathways that had an unadjusted *p*-value < 0.05 for the individual gene association with ovulatory years, as well as the individual components of ovulatory years (age at menopause [if postmenopausal] or age at diagnosis [if premenopausal], age at menarche, parity, and duration of oral contraceptive use). All analyses were conducted on all cases and then separately for women who were premenopausal or postmenopausal at the time of diagnosis.

## Results

Average age at diagnosis was 63 years with 78% of women being postmenopausal at diagnosis (Table 1). The median year at diagnosis was 2004; most patients (90%) were diagnosed with high-grade serous or poorly differentiated histologic subtypes and at stage III or IV (75%). The average lifetime ovulatory years was 36 years (SD = 6).

**Table 1:** Patient and tumor characteristics in NHS, NHSII, and NEC studies (*n* = 234)

Study, n (%)	
NEC	83 (35)
NHS	108 (46)
NHSII	43 (18)
Age at diagnosis, years, mean (SD)	63 (10)
Calendar year at diagnosis, median	2004
Menopausal status, n (%)	
Premenopausal	51 (22)
Postmenopausal	183 (78)
Age at menarche, years, mean (SD)	13 (1)
Oral contraceptive use, years, mean (SD)	3 (4)
Parity, mean (SD)	2 (2)
Age at menopause, years, mean (SD)^a^	50 (3)
Histotype, n (%)	
High grade serous/poorly differentiated	211 (90)
High grade endometrioid	22 (9)
Brenner	1 (1)
Stage, n (%)	
I	35 (15)
II	22 (9)
III	156 (67)
IV	19 (8)
Unknown	2 (1)
Ovulatory years, mean (SD)	36 (6)

*Abbreviations: NHS* Nurses’ Health Study, *NHSII* Nurses’ Health Study II, *NEC* New England Case–Control Study, *SD* Standard Deviation

^a^Among postmenopausal women

When examining expression of individual genes, none were significantly associated with lifetime ovulatory years after multiple testing correction (FDR > 0.19; Supplementary Table S1). However, when we examined biological pathways using GSEA, 18 pathways were significantly associated with lifetime ovulatory years at an FDR < 1.0 × 10^–5^ across all cases (Table 2). Higher ovulatory years were associated with downregulation of genes in pathways related to inflammation/immune function, including interferon gamma response (NES = -2.67, FDR = 3.80 × 10^–19^), interferon alpha response (NES = -2.52, FDR = 1.93 × 10^–11^), and inflammatory response (NES = -2.27, FDR = 3.99 × 10^–10^). Similar downregulation of genes related to cell proliferation pathways, such as E2F targets (NES = -2.62, FDR = 1.41 × 10^–18^) and G2M checkpoint pathways (NES = -2.48, FDR = 2.25 × 10^–15^), was also observed for greater lifetime ovulatory years.

**Table 2:** Significant pathways associated with lifetime ovulatory years with adjusted *p*-value < 1.0 × 10^–5^ in type II ovarian cancer tumor tissue in NHS, NHSII, and NEC (*n* = 234)

Pathway names	Database	Number of genes	NES	Unadjusted *p*-value	FDR
INTERFERON GAMMA RESPONSE	Hallmarks of cancer	184	-2.67	7.60 × 10^–21^	3.80 × 10^–19^
E2F TARGETS	Hallmarks of cancer	196	-2.62	5.62 × 10^–20^	1.41 × 10^–18^
G2M CHECKPOINT	Hallmarks of cancer	192	-2.48	1.35 × 10^–16^	2.25 × 10^–15^
INTERFERON ALPHA RESPONSE	Hallmarks of cancer	92	-2.52	1.54 × 10^–12^	1.93 × 10^–11^
CELL CYCLE CHECKPOINTS	REACTOME	253	-2.27	8.90 × 10^–14^	3.78 × 10^–11^
CELL CYCLE MITOTIC	REACTOME	477	-1.97	6.79 × 10^–14^	3.78 × 10^–11^
INFLAMMATORY RESPONSE	Hallmarks of cancer	153	-2.27	3.99 × 10^–11^	3.99 × 10^–10^
TNFA SIGNALING VIA NFKB	Hallmarks of cancer	176	-2.17	1.33 × 10^–10^	1.11 × 10^–9^
ALLOGRAFT REJECTION	Hallmarks of cancer	142	-2.17	9.30 × 10^–10^	6.64 × 10^–9^
DNA REPLICATION	REACTOME	124	-2.36	6.56 × 10^–11^	1.86 × 10^–8^
SEPARATION OF SISTER CHROMATIDS	REACTOME	176	-2.09	9.53 × 10^–10^	2.02 × 10^–7^
MITOTIC METAPHASE AND ANAPHASE	REACTOME	221	-2.04	2.87 × 10^–9^	4.06 × 10^–7^
S PHASE	REACTOME	157	-2.13	2.41 × 10^–9^	4.06 × 10^–7^
OXIDATIVE PHOSPHORYLATION	KEGG	112	-2.18	6.59 × 10^–9^	1.05 × 10^–6^
G2 M CHECKPOINTS	REACTOME	131	-2.18	1.38 × 10^–8^	1.67 × 10^–6^
M PHASE	REACTOME	338	-1.83	1.83 × 10^–8^	1.94 × 10^–6^
HYPOXIA	Hallmarks of cancer	168	-1.85	8.98 × 10^–7^	5.62 × 10^–6^
THE CITRIC ACID TCA CYCLE AND RESPIRATORY ELECTRON TRANSPORT	REACTOME	161	-2.01	9.21 × 10^–08^	8.69 × 10^–06^

Type II ovarian cancer tumors include high-grade serous, poorly-differentiated, and high-grade endometrioid tumors

*Abbreviations: FDR* False Discovery Rate, *NHS* Nurses’ Health Study, *NHSII* Nurses’ Health Study II, *NEC* New England Case–Control Study, *NES* Normalized enrichment score

**Fig. 1:**
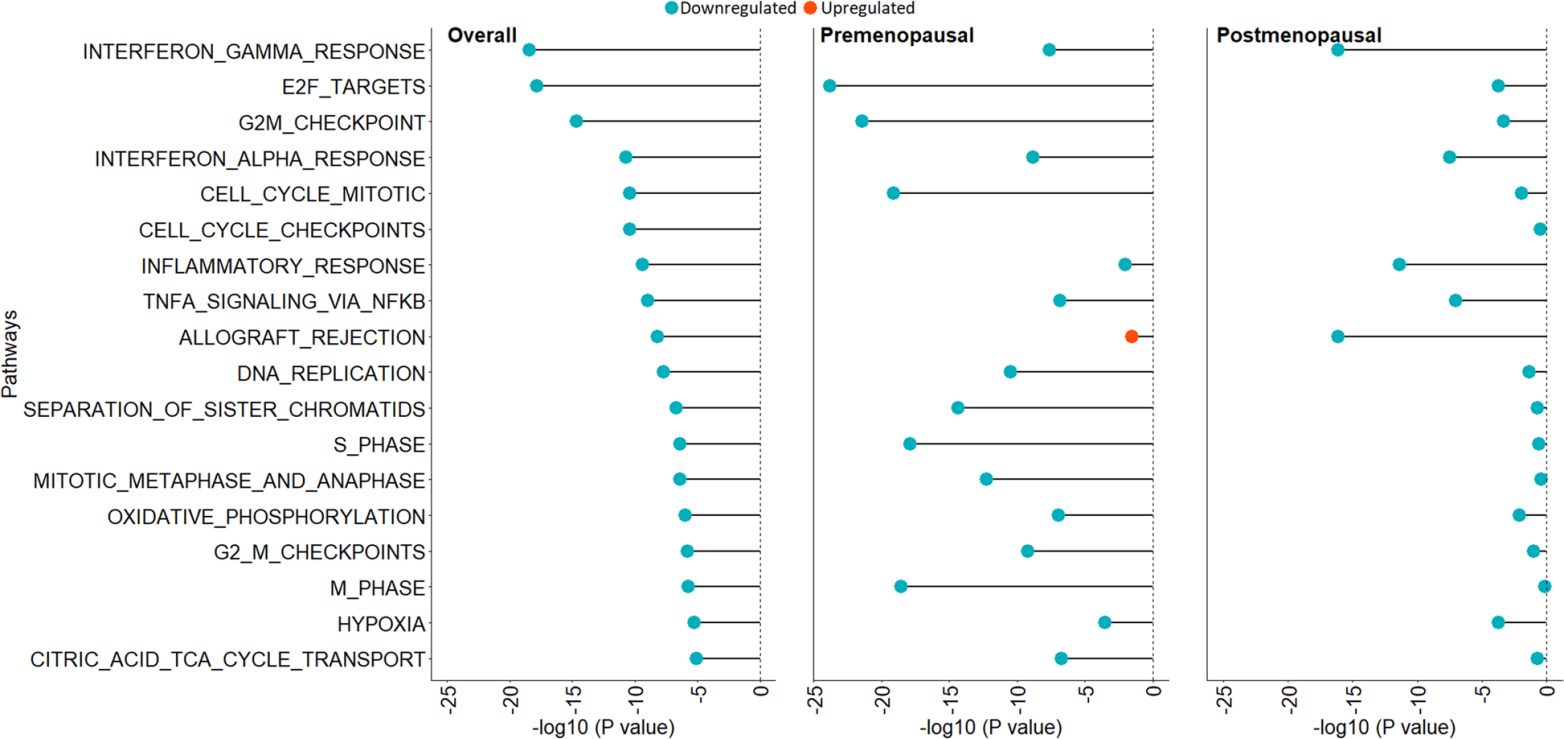
Significant pathways associated with lifetime ovulatory years overall and stratified by menopausal status with FDR < 1.0 × 10^–5^ in type II ovarian cancer tumor tissue in NHS, NHSII, and NEC studies (*n* = 234). Pathways associated with lifetime ovulatory years overall are presented in the order of statistical significance in the left figure including all tumors (adjusted p-value plotted on the x axis). Results of the same pathways when restricted to premenopausal and postmenopausal cases are presented in the middle and right figures, respectively. Upregulated pathways are denoted by red bubbles and downregulated pathways are denoted by blue bubbles. Cell cycle checkpoints pathway was removed in premenopausal women due to NES and p-values not calculated. Of note, “G2M_CHECKPOINT” is from Hallmarks of Cancer database and “G2_M_CHECKPOINTS” is from Reactome database

We then examined associations of these pathways separately for premenopausal and postmenopausal women at diagnosis (Fig. 1). Among premenopausal women, qualitatively stronger downregulation of genes related to cell proliferation such as E2F targets (NES = -3.01, FDR = 1.53 × 10^–24^) and G2M checkpoint pathways (NES = -2.90, FDR = 3.50 × 10^–22^) in relation to higher lifetime ovulatory years was observed compared to postmenopausal women (E2F targets [NES = -1.73, FDR = 0.0002] and G2M checkpoint pathways [NES = -1.71, FDR = 0.0005]). Conversely, in postmenopausal women, stronger downregulation of inflammatory response pathways was observed (e.g., interferon gamma response pathway [NES = -2.58, FDR = 7.81 × 10^–17^] and inflammatory response pathway [NES = -2.43, FDR = 4.31 × 10^–12^]) than in premenopausal women (interferon gamma response pathway [NES = -2.03, FDR = 2.38 × 10^–8^] and inflammatory response pathway [NES = -1.44, FDR = 0.009]).

**Fig. 2:**
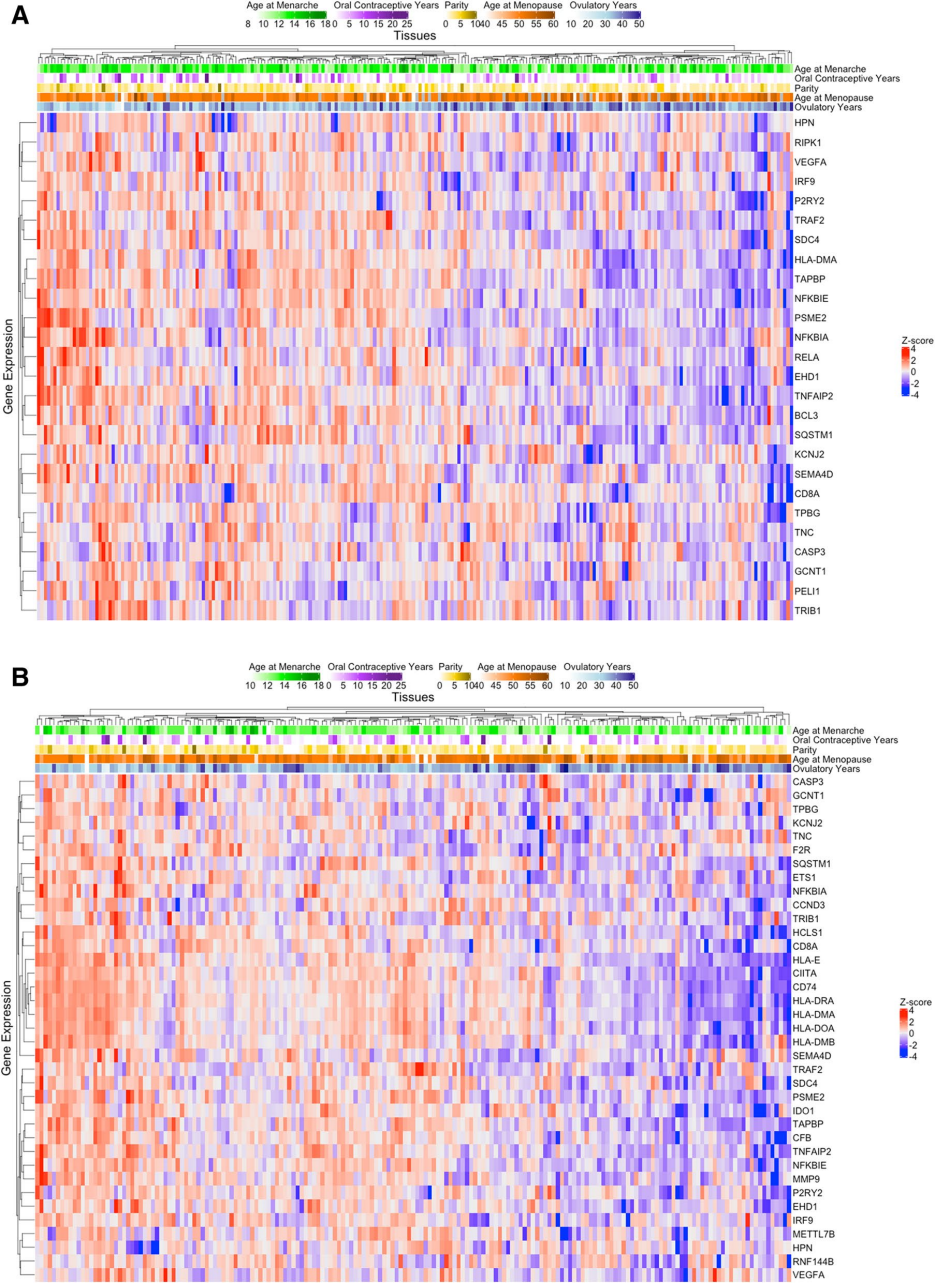
Heatmap of genes associated with ovulatory years and its individual components in significantly associated inflammation/immune-related pathways. Of the genes included in the significant pathways (i.e., interferon gamma response, interferon alpha response, inflammatory response, TNAa signaling via NFkB, allograft rejection), individual genes associated with ovulatory years with unadjusted *p*-value < 0.05 were included in the heatmap. The top of the heatmap shows values for age at menarche (green), years of oral contraceptive use (purple), parity (yellow), age at menopause (orange; gray bars indicate premenopausal cases), and the summary ovulatory years (blue). **A** Heatmap including all type II ovarian cancer tumors in NHS/NHSII/NEC (*n* = 234) and **B** Heatmap restricting to type II ovarian cancer tumors from patients who were postmenopausal at time of diagnosis (*n* = 183)

Multiple genes overlapped across pathways (Supplementary Figure S3), thus, we assessed the gene expression pattern for individuals genes within the significant inflammation/immune pathways (i.e. interferon gamma response, interferon alpha response, inflammatory response, TNAα signaling via nuclear factor-kappa B (NFkB), allograft rejection) that had an unadjusted *p*-value < 0.05 for the individual gene association with ovulatory years (Fig. 2A). This included genes in the NFkB pathway (e.g., NKFBIA, NKFBIE, TRAF2, RELA), which were downregulated among ovarian tumors from patients with greater lifetime ovulatory years. Interestingly, ovarian tumors from patients with shorter duration of oral contraceptive use tended to cluster with inflammation/immune related genes being downregulated. Similar trends were observed when restricting to postmenopausal women at diagnosis (Fig. 2B), although multiple genes related to major histocompatibility complex (MHC) class II were nominally significant among this population (e.g., CIITA, CD74, HLA-DRA, HLA-DMA, HLA-DOA, HLA-DMB), suggesting pathways related to adaptive immune response may be downregulated.

**Fig. 3:**
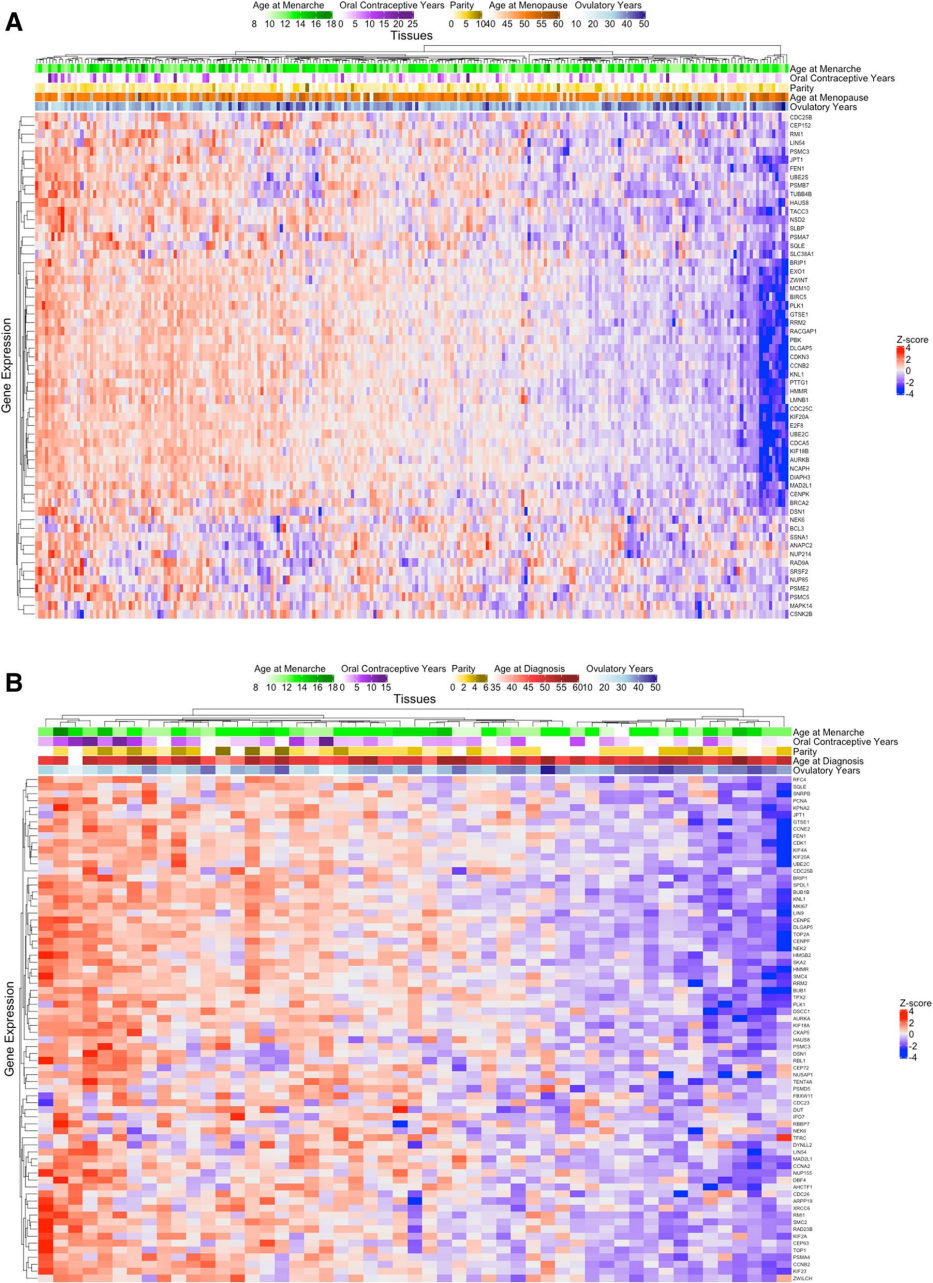
Heatmap of genes associated with ovulatory years in the proliferation-related pathways. Of the genes included in the significant pathways (i.e.E2F targets, G2M checkpoint, cell cycle checkpoints, cell cycle mitotic, DNA replication, separation of sister chromatids, mitotic metaphase and anaphase, S phase, G2 M checkpoints, M phase), individual genes associated with ovulatory years with unadjusted *p*-value < 0.05 were included in the heatmap. **A** Heatmap including all type II ovarian cancer tumors in NHS/NHSII/NEC (*n* = 234) and **B** Heatmap restricting to type II ovarian cancer tumors from patients who were premenopausal at time of diagnosis in NHS/NHSII/NEC (*n* = 51). Grey bars in age at menopause indicate premenopausal cases

We also looked at genes (unadjusted *p*-value < 0.05) in the significant proliferation-related pathways (i.e., E2F targets, G2M checkpoint, cell cycle checkpoints, cell cycle mitotic, DNA replication, separation of sister chromatids, mitotic metaphase and anaphase, S phase, G2 M checkpoints, M phase) for all women and premenopausal cases (Fig. 3A, B). This included multiple genes related to DNA damage repair (e.g., BRCA2, BRIP1, RMI1, TACC3, EXO1), which were downregulated with higher ovulatory years overall. Patients with shorter duration of oral contraceptive use tended to have downregulation of these proliferation-related genes, especially when restricted to premenopausal women, although the sample size was limited.

## Discussion

Using data from three large studies, we examined ovarian tumor gene expression profiles associated with lifetime ovulatory years leveraging whole exome RNA sequencing. While we did not observe individual genes that were differentially expressed by lifetime ovulatory years after multiple testing correction, there were several significant annotated biological pathways. Interestingly, increasing lifetime ovulatory years were associated with downregulation of inflammation/immune and cell proliferation pathways. Furthermore, when stratified by menopausal status, downregulation of cell proliferation pathways, such as E2F targets and G2M checkpoint pathways, were suggestively more strongly associated with greater lifetime ovulatory years among premenopausal patients whereas inflammation/immune-related pathways, such as interferon gamma response, were suggestively more strongly downregulated among postmenopausal women.

One mechanism by which greater lifetime ovulatory cycles are thought to be associated with increased ovarian cancer risk is through increasing local inflammation [12, 35] at the ovary and fallopian tube during and after ovulation, as there are many pro-inflammatory factors in the follicular fluid released during this process [36] as well as inflammatory processes associated with epithelial wound healing [37]. Thus, our results observing downregulation of inflammatory/immune related pathways in tumors among women who had higher lifetime ovulatory years was unexpected. However, we have previously reported that increasing lifetime ovulatory years were associated with lower chronic systemic inflammation [38], which was primary driven by higher inflammatory markers (i.e., c-reactive protein, interleukin-6) among women with early menopause and past and current users of oral contraceptives, even among postmenopausal women. Thus, it is possible that while ovulation causes acute inflammatory events, the long term, cumulative effect of high numbers of ovulations (driven by limited or no oral contraceptive use and late menopause) and its associated reduction in systemic inflammation may then be associated with decreases in the pro-inflammatory local microenvironment in the ovarian tumor. Interestingly, the association in our study was stronger among postmenopausal women, who would have experienced longer periods of low or high inflammation based on their prior reproductive history. Notably, a pro-inflammatory tumor microenvironment has been reported to play a key role in tumor progression and associated with poor prognosis [39, 40], although prior studies have reported no clear associations between lifetime ovulatory years and ovarian cancer survival [41, 42].

Wound healing after ovulation initiates cell proliferation and therefore our results observing downregulation of cell proliferation pathways (e.g. E2F targets) with increasing ovulatory cycles was also unexpected. E2F target genes include transcription factors that are essential for cell proliferation and differentiation [43]. At the same time, E2F1, which is one of the E2F target genes, also induces apoptosis in ovarian, breast and prostate tumors [44, 45]. Therefore, it is possible that the observed downregulation of E2F target gene pathway is reflecting the downregulation of E2F1-related pathways, impairing apoptosis in the tumor. In addition, when examining individual genes in the significant cell proliferation pathways, multiple DNA repair genes were downregulated among women with greater lifetime ovulatory years. Consequently, dysfunction of these DNA damage repair genes could have led to accumulation of somatic mutations resulting in ovarian carcinogenesis. In this aspect, our results are in line with a prior study reporting greater number of lifetime ovulatory cycles were associated with increased amount of proliferation-associated DNA damage and increased risk of developing p53 positive ovarian cancer [3].

Interestingly, we observed different strengths of pathway associations when stratifying by menopausal status at diagnosis. In ovarian tumors from premenopausal patients (*N* = 51), pathways related to cell proliferation and DNA damage repair genes were more strongly associated with greater lifetime ovulatory years. Since premenopausal women are likely to be ovulating, it is possible that the acute local pro-inflammatory environment impairs the DNA repair process [46, 47]. Notably, chronic inflammation is associated with increased oxidative stress, which can increase DNA damage and genomic instability [47, 48]. Another possible explanation for this observation is the potential for having higher prevalence of BRCA mutation carriers in premenopausal patients. Several prior studies suggest that ovarian cancer cases with germline BRCA or homologous recombination gene mutations tend to be diagnosed at a younger age compared to those without [49, 50], although the reported average age at diagnosis for ovarian cancer among the germline mutation carriers in these studies was around 53 years, in which over half are presumably postmenopausal women. Moreover, in our study population where the participants are predominantly white, non-Ashkenazi Jewish population, we expect the prevalence of BRCA mutation carriers to be lower. Further investigations are needed to elucidate the intersections between greater lifetime ovulatory years and homologous recombination/DNA repair pathways in the ovarian tumor, especially in premenopausal patients. On the other hand, in ovarian tumors from postmenopausal patients (*N* = 183), downregulation of inflammation/immune-related pathways and genes related to MHC class II were strongly associated with greater lifetime ovulatory years. MHC class II molecules are mostly expressed by B cells, macrophages, and dendritic cells and activate naïve CD4 + T cells, but can also be expressed by tumor cells [51, 52], which may enhance the ability of the immune system to recognize the tumor [51, 53]. Therefore, downregulation of MHS class II genes may result in decreased eradication of tumor cells, leading to development of ovarian cancer [53, 54]. In fact, increased MHC class II expression in advanced stage serous ovarian cancers have been associated with improved survival [52]. These results suggest ovulatory years may influence ovarian carcinogenesis through potentially different biologic pathways in premenopausal women who are still ovulating versus postmenopausal women.

Our study has several strengths. We were able to conduct a genome-wide transcriptomics analysis using RNA sequencing data, allowing a comprehensive evaluation of genes and pathways related to lifetime ovulatory years in a large number of highly annotated, type II tumors. In our analyses, we did not observe statistically significant individual genes although we identified multiple biological pathways associated with ovulatory years that were highly statistically significant. Pathway analysis may have been able to detect relevant biological differences more robustly compared to single gene analysis with our limited sample size for several reasons. First, biological processes often affect groups of genes that share common biological function and this may be identified better in a pathway analysis [33]. Also, if multiple genes from a common pathway (e.g., DNA repair genes) are all modestly altered, pathway analysis has increased power to detect an association over an individual gene analysis. One limitation of our study is that we did not have an independent validation dataset. However, to our knowledge this is the first study to report gene expression profiles associated with lifetime ovulatory years in ovarian tumors and with our large sample size and observing similar pathways being significantly associated across three different biologic pathway databases reduces the likelihood of false positives. Another key strength of this study is the ability to link epidemiologic information with tumor features. Majority of the cases in this study were non-Hispanic white and therefore studies in diverse population is warranted to validate our findings.

## Conclusions

In summary, we identified several biological pathways, particularly related to downregulation of inflammation/immune processes and cell proliferation/DNA repair, in ovarian tumors that were significantly associated with higher lifetime ovulatory years, with potentially different biological pathways being more important in premenopausal women who are still ovulating versus postmenopausal women. Further epidemiological and mechanistic research is warranted to validate our finding in independent datasets, expand the investigation to include other histologic subtypes, and incorporate additional biologic data (e.g., somatic mutation, methylation data). Our results provide novel insight into the possible underlying biological mechanism of how long-term exposure to ovulation impacts ovarian carcinogenesis that may lead to development of targeted prevention strategies in the future.

## Supplementary Information

**Additional file 1: Supplementary Table S1.** Association between individual gene expression and ovulatory years.

**Additional file 2: Supplementary Figure S1.** 

**Additional file 3: Supplementary Figure 2.**

**Additional file 4: Supplementary Figure 3.**

## Data Availability

Data in this study are not publicly available due to participant confidentiality and appropriate adherence to ethical requirements. Further information including the procedures to obtain and access data from the Nurses’ Health Studies is described at https://www.nurseshealthstudy.org/researchers (contact email: nhsaccess@channing.harvard.edu). The NEC data that support the findings of this study are available upon request and review by study leadership.
