# Supplementary figures and images for: Lifetime ovulatory years and ovarian cancer gene expression profiles
Source: J Ovarian Res. 2022 May 13;15:59. doi: 10.1186/s13048-022-00995-1 (PMC9102743; doi:10.1186/s13048-022-00995-1)

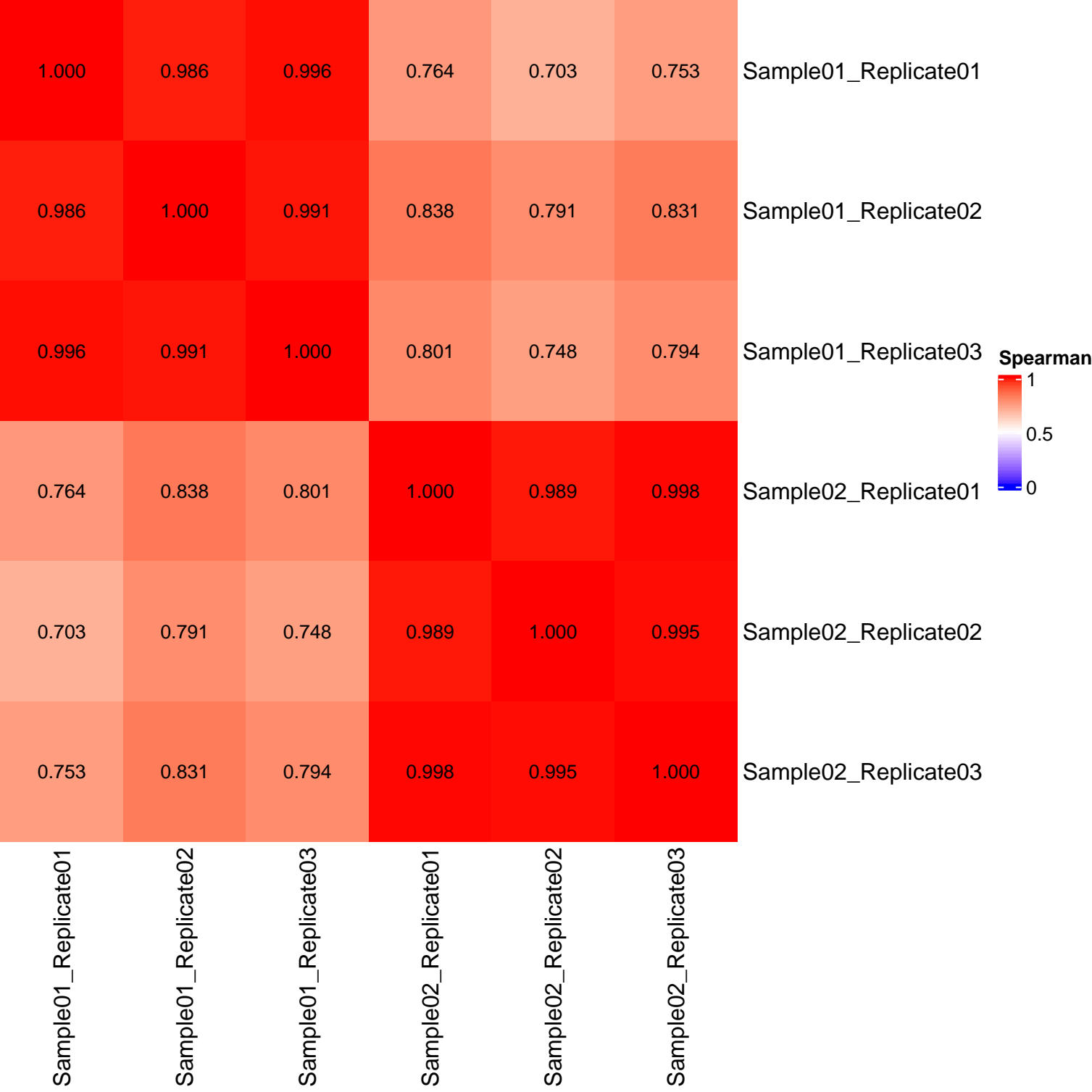

Supplement: Supplementary file 2 — Additional file 2: Supplementary Figure S1. [file 13048_2022_995_MOESM2_ESM.pdf]

Supplementary Figure 1

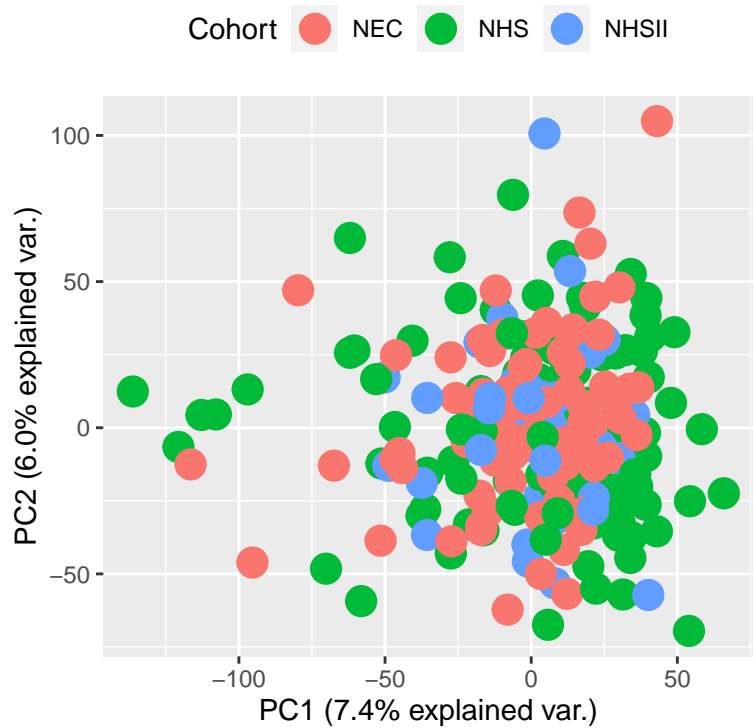

Supplement: Supplementary file 3 — Additional file 3: Supplementary Figure 2. [file 13048_2022_995_MOESM3_ESM.pdf]

Supplementary Figure 2

Pathways

Genes

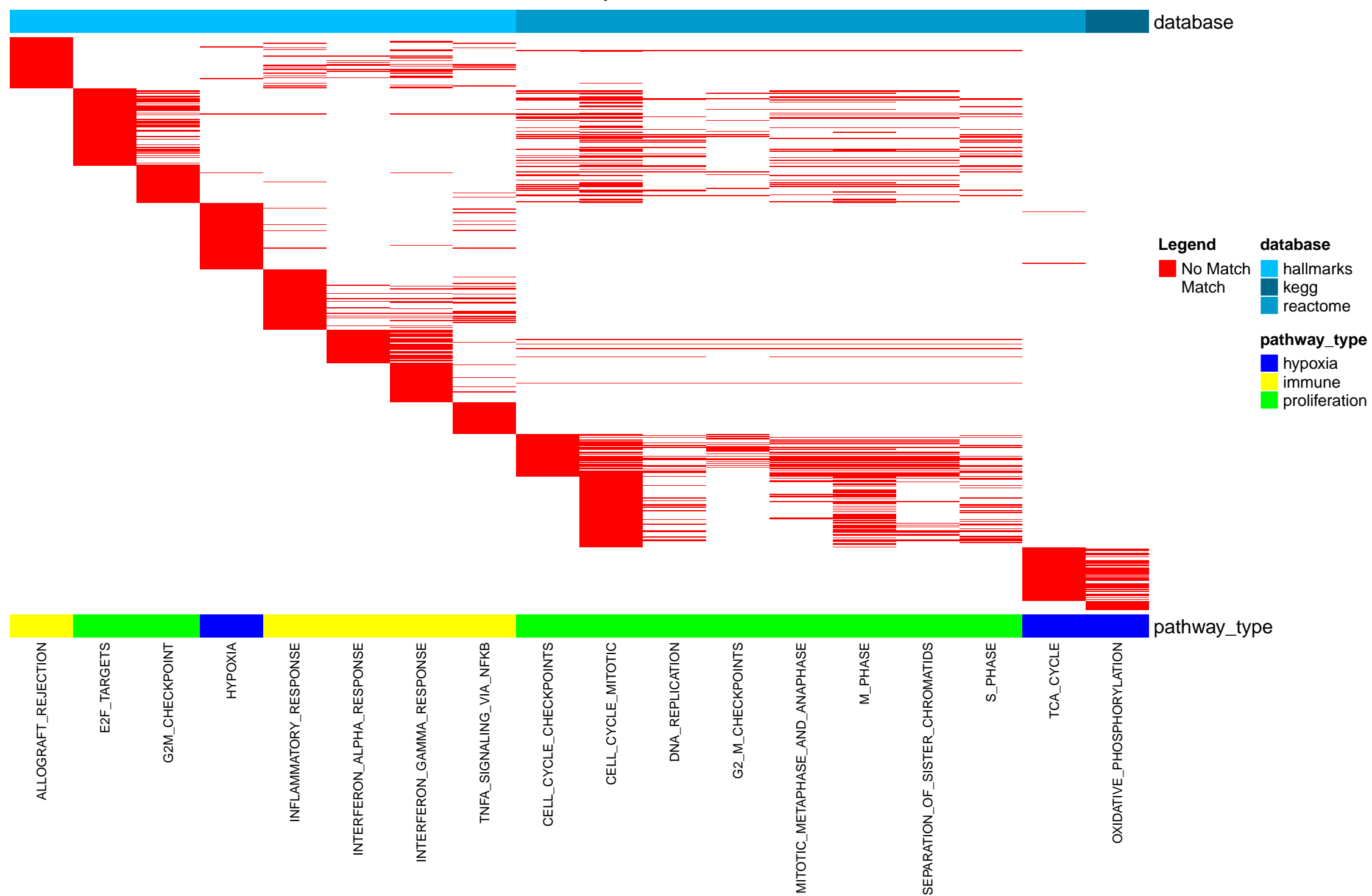

Supplement: Supplementary file 4 — Additional file 4: Supplementary Figure 3. [file 13048_2022_995_MOESM4_ESM.pdf]
